# Corticospinal Tract Integrity and Long-Term Hand Function Prognosis in Patients With Stroke

**DOI:** 10.3389/fneur.2019.00374

**Published:** 2019-04-15

**Authors:** Yeun Jie Yoo, Jae Won Kim, Joon Sung Kim, Bo Young Hong, Kyoung Bo Lee, Seong Hoon Lim

**Affiliations:** ^1^Department of Rehabilitation Medicine, Yeouido St. Mary's Hospital, College of Medicine, The Catholic University of Korea, Seoul, South Korea; ^2^Department of Rehabilitation Medicine, St. Vincent's Hospital, College of Medicine, The Catholic University of Korea, Seoul, South Korea

**Keywords:** stroke, stroke rehabilitation, corticospinal tract, diffusion tensor imaging, hand function, prognosis

## Abstract

**Background:** The restoration of hand function is an important goal for patients with stroke. This study investigated the relationship between corticospinal tract (CST) integrity and the functional status of the hand in patients with stroke 6 months after onset and evaluated which of the following values would be useful for predicting hand function: fiber number (FN), fractional anisotropy (FA) at the mid-pons, and FA at the pontomedullary junction.

**Methods:** The present retrospective cross-sectional observational study assessed 44 patients with stroke who were able to walk without using a walking aid or orthosis. The final hand function results were classified into three groups: no recovery (unable to grasp), partial recovery (able to grasp, unable to oppose), and full recovery (able to grasp and oppose). All subjects underwent diffusion tensor imaging (DTI) at 6 months after stroke onset. Values for FA at the mid-pons and pontomedullary junction and CST FN were measured. The normalization ratio for FN and FA was calculated using the following formula: data for affected hand/data for non-affected hand.

**Results:** The normalized FN, FA (mid-pons), and FA (pontomedullary junction) DTI values differed significantly. The FA (mid-pons) value for the full recovery group was higher than those for the other groups. The FA (mid-pons) value for the partial recovery group was higher than that for the no recovery group. The normalized FA (mid-pons) value differed significantly among all three groups.

**Conclusions:** The present study showed that CST integrity (at 6 months after onset) in patients with chronic stroke was related to functional hand status. In addition, the mid-pons FA value was more predictive of functional restoration of the hand than the FN or FA value at the pontomedullary junction. These results may be useful in predicting the functional restoration of the hand and understanding the functional prognosis of stroke.

## Introduction

Restoration of hand function is one of the most important goals for patients with stroke (1). Thus, techniques that aid in predicting restoration of hand function are also important for clinicians. Clinicians often predict the prognosis for motor function of an upper or lower limb based on the size and location of brain lesions (2–7). Clinicians have also used motor ability at an early stage or at discharge to predict the long-term motor outcome (1, 8). In addition, the relationship between corticospinal tract (CST) integrity and motor function has been evaluated in patients with stroke (9, 10).

The evaluation of CST integrity using diffusion tensor imaging (DTI) has been explored as a technique for predicting functional motor prognosis in patients with stroke (11–13). The CST injury seen using axial diffusivity in the acute phase may predict upper-limb strength (11). In addition, information about the early integrity of the CST may be useful for predicting long-term motor outcomes, specifically motor recovery of the upper extremity and hand (13). One study showed that the presence of both motor evoked potentials and a preserved CST may predict recovery of ambulation (12). However, in that study, the correlation between the corticospinal pathway and ambulation was not as strong as that between CST and hand function (14). It has been suggested that the cortico-reticular pathway (CRP), rather than the CST, plays the major role in ambulation (15). Gait has been shown to depend not only on the CST but also on the CRP, via the relationship of increased muscle tone with vestibular function (16, 17). Thus, interest in the effect of the CST on motor function focuses on the function of the upper limb, especially the hand. Recent research has shown that the recovery of motor function in the affected hand after stroke is related to excitability, size, volume, and location (18, 19).

We hypothesized that CST integrity would be reflected in the functional recovery of motor function, especially upper limb function compared with lower limb function (11, 13, 20, 21) because the CST is primarily involved in fine motor skills such as hand function (22). However, DTI for CST did not discriminate CST of the upper limb from the CST of the lower limb. We restricted the enrolled patients with a functionally recovered lower-limb, had functionally different state of the hand. The primary aim of this study was to investigate the relationship between CST integrity and the functional status of the hand in patients with chronic stroke. A secondary aim was to identify which DTI data would be useful for predicting hand function from among fiber number, fractional anisotropy (mid-pons), and fractional anisotropy (pontomedullary junction).

## Materials and Methods

### Study Design and Participants

This was a retrospective cross-sectional observational clinical trial. Forty-four right handed subjects with stroke were recruited from the Department of Rehabilitation Medicine, St. Vincent's Hospital, Suwon, South Korea between August 2016 and July 2018. All of the subjects had suffered first-ever supratentorial unilateral stroke, and all met the following criteria: (1) first-ever unilateral stroke; (2) ability to follow verbal instructions; (3) Fugl–Meyer Assessment score lower than 90 (5, 7); (4) ability to walk independently without an orthosis or walking aid at 6 months after stroke onset; and (5) magnetic resonance imaging (MRI) scan at 6 months ± 14 days after onset. Exclusion criteria were (1) brain surgery during the period of observation, (2) history of inflammatory arthritis or inflammatory myopathy, and (3) underlying degenerative brain disease, such as Parkinson's disease. Considering the maturation of neurological recovery, a time of 6 months after stroke onset was defined as the completion of neurological recovery (23).

All subjects provided demographic, clinical, and brain MRI data. The brain MRI (DTI) was performed at 6 months ± 14 days after stroke onset, at the time of functional evaluation. The results of the hand function evaluation were classified into three groups: no recovery (unable to grasp), partial recovery (able to grasp, unable to oppose), and full recovery (able to grasp and oppose). We defined full recovery as restoration of ability in terms of power grasp, opposition, lateral pinch, and tip pinch, which would enable the use of a pencil for writing and of chopsticks for eating. Partial recovery was defined as the ability to grasp and release, but not perform delicate hand functions, such as opposition, lateral pinch, and tip pinch. No recovery was defined as inability to perform full grasp. All subjects received routine physical and occupational therapy for 1–2 h per day, 5 days per week. They also received speech therapy as needed. When the patients were fully recovered, rehabilitation therapy was stopped at a time determined at a team conference that included a physiatrist, therapists, and the patients themselves. The rehabilitation program for all subjects began within 7 days after onset and continued until 6 months after onset.

Because the CST cannot distinguish CST of the upper limb from CST of the lower limb, we enrolled the subjects that had relatively same functional levels of lower limbs. All included subjects had gotten hemiplegia due to stroke at acute phase, confirmed by clinical symptom and MRI scan, were took the neurologic examination and MRI scan at 6 months after onset.

Because this study was a cross-sectional observational study examining the relationship between DTI and hand function, the sample size was not calculated beforehand. However, sample sizes in previous studies have ranged from 22 to 33 (21, 24); the sample size for this study was set at more than 33 subjects. A total of 263 patients were initially enrolled during the recruitment period; however, 131 patients were excluded due to their inability to walk without a walking aid or orthosis at 6 months after onset, 17 were excluded for a subsequent attack or other complication within 6 months, 47 were excluded due to missing 6-month MRI data, and 24 were excluded due to attrition, such as transfer to another rehabilitation center at a distant location.

The study protocol was reviewed and approved by the Institutional Review Board of Catholic University, College of Medicine (Registry No. VC18RESI0048); the need for informed consent was waived by the board.

## DTI Acquisition

DTI was performed using a 3.0-T MRI (MAGNETOM® Verio, Siemens, Erlangen, Germany) equipped with a six-channel head coil. The data were acquired in the form of single-shot spin-echo echo-planar images, with axial slices covering the whole brain across 76 interleaved slices of 2.0 mm thickness (no gap); repetition time (TR)/echo time (TE) = 14,300/84 ms; field of view = 224 × 224 mm^2^; matrix 224 × 224; voxel size 1 × 1 × 2 mm^3^; number of excitations = 1. Diffusion sensitizing gradients were applied in 64 noncollinear directions with a b-value of 1,000 ms/mm^2^. The b = 0 images were scanned before acquisition of the diffusion-weighted images, with 65 volumes in total (24).

### Image Processing and Diffusion Tensor Tractography

**Figure 1:**
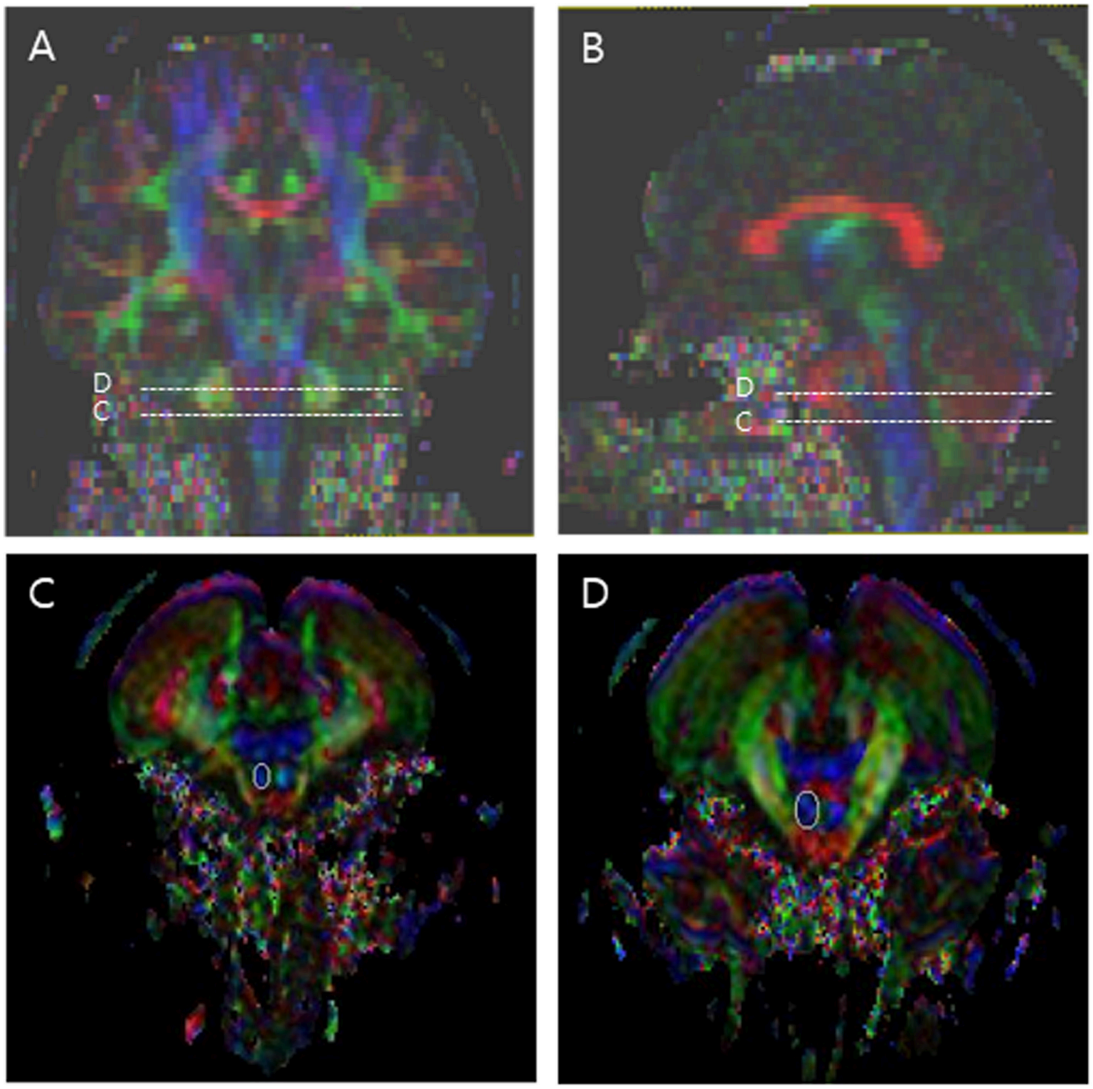
Regions of interest (ROIs) used to reconstruct the corticospinal tract (CST) on diffusion tensor imaging. The stippled lines indicate the ROIs. The solid lines indicate the seed ROI for the corticospinal tract. **(A)** Coronal color FA map; **(B)** Sagittal color FA map; **(C)** The seed ROI of CST at the pontomedullary junction; **(D)** The seed ROI of CST at the mid-pons.

Fiber tracking was based on the fiber assignment continuous tracking (FACT) algorithm and a multiple regions of interest (ROIs) approach using DTI-Studio (10, 25). Two ROIs were used for CST reconstruction. The first seed ROI was placed on the pontomedullary junction portion of the CST, and the second on the mid-pons portion of the CST (Figure 1) (26, 27). The termination criteria were fractional anisotropy (FA) < 0.2 and an angle change of >60 degrees (10). The CST fiber number (FN) and the FA value in both ROIs were measured in both hemispheres of all patients. The normalization ratio for FN and FA values was calculated using the following formula: data of affected hand/data of non-affected hand (10). After then, we analyzed the values; normalized FN, FA at mid-pons, FA at pontomedullary junction value.

### Statistical Analysis

The CST FN, FA (mid-pons), and FA (pontomedullary junction) values in the three groups are presented as median (interquartile range: first–third quartiles). The distributions of patients according to stroke side and type were tested by Pearson's chi-square test. Differences among the three groups were evaluated using the Kruskal–Wallis test, followed by the Mann–Whitney *U*-test with the Bonferroni correction. The Kruskal–Wallis test was two-tailed, and *p*-values < 0.05 were deemed significant. The Mann–Whitney *U*-test with the Bonferroni correction were two-tailed, and *p*-values ≤ 0.0166 were deemed to be significant. All statistical analyses were performed using SPSS software for Windows (ver. 21.0; SPSS, Chicago, IL, USA).

## Results

The demographic and clinical characteristics of the three groups are shown in Table 1. The distributions of stroke side and type did not differ among the groups.

**Table 1:** Participants' demographic data.

	**Full recovery group (*n* = 16)**	**Partial recovery group (*n* = 13)**	**No recovery group (*n* = 13)**	***P-*value**
Age, years	57.5 (49.0–62.8)	52.0 (44.0–65.5)	55.0 (48.5–65.0)	
Female sex	10 (62.5)/6(37.5)	8 (62.5)/5 (28.5)	9 (69.2)/4 (30.8)	1,000
Right-handed	16 (100)	13 (100)	13 (100)	1.000
Left hemispheric lesion	10 (62.5)	10 (76.9)	11 (84.6)	0.380
Stroke type		–		0.626
Hemorrhage	8 (50.0)	9 (69.2)	8 (61.5)	
Infarction	8 (50.0)	4 (30.8)	5 (38.5)	
Brain injury location		–		0.233
Cortex	2 (12.5)			
Subcortex	8 (50.0)	10 (76.9)	6 (46.2)	
Mixed (cortex and subcortex)	6 (37.5)	3 (23.1)	7 (53.8)	

*Values are the median (range: 25th−75th percentile), or n (%), unless otherwise indicated. P-values were tested using Pearson's chi-square test*.

**Figure 2:**
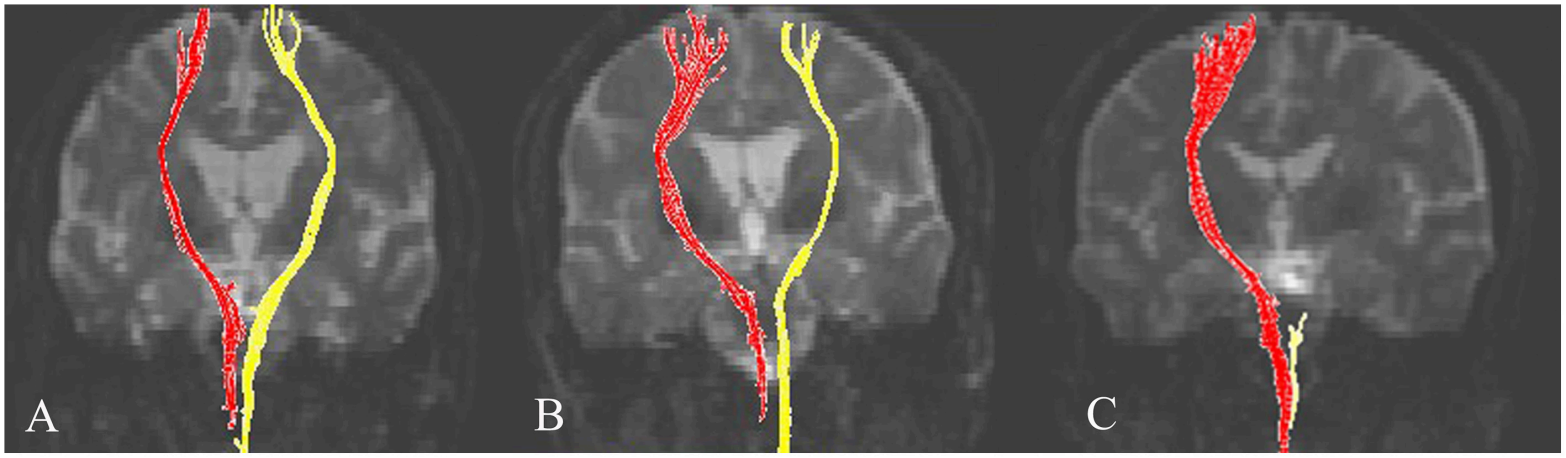
Representative diffusion tensor tractography images of the corticospinal tract in typical subjects from the three groups. Subjects from the **(A)** full recovery, **(B)** partial recovery, and **(C)** no recovery groups. The non-affected tract is shown in red, and the affected tract in yellow.

Representative DTIs of typical subjects from the three groups are shown Figure 2.

**Figure 3:**
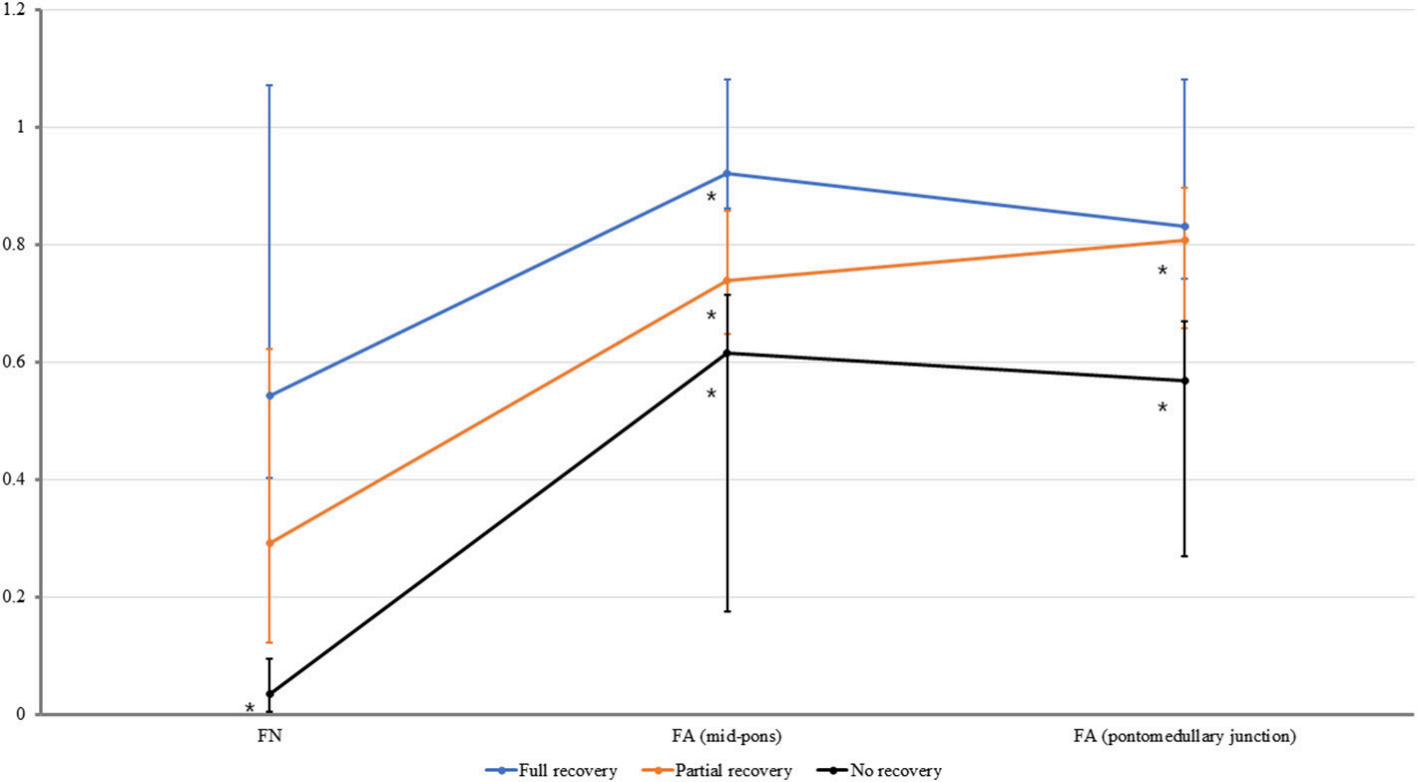
The normalized fiber number (FN), fractional anisotropy (FA) at the mid-pons, and FA at the pontomedullary junction of the corticospinal tract in all groups. The median values are shown as dots. For all values, the error bar shows the interquartile range between the first and third quartiles. *The FN value for no recovery group was lower than those that for full recovery group (*P* ≤ 0.0166). *The FA (mid-pons) value for the full recovery group was higher than those for the other two groups (*P* ≤ 0.0166), and the FA (mid-pons) value for the partial recovery group was higher than that for the no recovery group (*P* ≤ 0.0166). *The FA (pontomedullary junction) value for the full recovery group and partial recovery group were higher than that for the no recovery group (*P* ≤ 0.0166, respectively). The FA (pontomedullary junction) value for the full recovery group and that for partial recovery group were not significantly different.

The FN, FA (mid-pons), and FA (pontomedullary junction) values of the CST for all three groups are presented in Table 2 and Figure 3. Significant differences were found in the normalized FN, FA (mid-pons), and FA (pontomedullary junction) DTI values (Table 2). The normalized FN, FA (mid-pons), and FA (pontomedullary junction) values differed significantly among three groups. The FN and FA (pontomedullary junction) value of all groups did not be shown significant difference between sub-two groups. The FA (mid-pons) value for the full recovery group was higher than those for the other two groups, and the FA (mid-pons) value for the partial recovery group was higher than that for the no recovery group.

**Table 2:** FN, FA (mid-pons), and FA (pontomedullary junction) values of the CST by group.

	**Full recovery**	***P*^**1**^**	**Partial recovery**	***P*^**2**^**	**No recovery**	***P*^**3**^**
Normalized FN	0.54^*^ (0.40–1.07)	0.022	0.29^*^ (0.12–0.62)	0.017	0.03^*^ (0.00–0.09)	0.000
Normalized FA (Mid-pons)	0.92^*^ (0.86–1.08)	0.000	0.74^*^ (0.64–0.85)	0.009	0.62^*^ (0.17–0.71)	0.000
Normalized FA (pontomedullary junction)	0.83^*^ (0.74–1.07)	0.136	0.81^*^ (0.66–0.89)	0.001	0.57^*^ (0.24–0.66)	0.000

*Values are the median (interquartile range: first–third quartiles), and normalized values (affected/non-affected)*.

*CST, corticospinal tract; FA, fractional anisotropy; FN, fiber number*.

* *P < 0.05. The three groups were compared using the Kruskal–Wallis test*.

*P^1^ Comparison between full and partial recovery groups with the Mann–Whitney U-test with the Bonferroni correction (P ≤ 0.0166 deem to be significant)*.

*P^2^ Comparison between partial and no recovery groups with the Mann–Whitney U-test with the Bonferroni correction (P ≤ 0.0166 deem to be significant)*.

*P^3^ Comparison between no and full recovery groups with the Mann–Whitney U-test with the Bonferroni correction (P ≤ 0.0166 deem to be significant)*.

## Discussion

Previous studies have demonstrated the clinical usefulness of DTI for evaluating function in patients with stroke. The results of the present study elaborated further on those studies, showing that CST integrity at 6 months after onset in patients with chronic stroke was reflected in the functional status of the affected hand. In addition, the mid-pons FA values were more predictive of hand function than were the pontomedullary junction FN or FA values. The DTI CST images were evaluated several times in terms of their relationship to hand function (10, 11, 13, 20, 21). A previous study found that, in patients with a corona radiata infarct, the integrity of the CST, as assessed by DTI obtained during the early stage, appeared to be helpful in predicting motor outcomes on the affected side (20). Most previous reports have shown that damage to the CST is useful for predicting restoration of motor function in patients with stroke (20, 21, 27). We hypothesized that the normalized FA and FN values in a functionally recovered state would reflect a hand function status similar to that of chronic stroke patients of the same functional state. We defined full functional recovery as occurring at 6 months after onset (23). Consistent with our hypothesis, CST integrity at 6 months after onset reflected the functional status of the affected hand at 6 months, i.e., a nearly fully recovered functional state.

The effects of change of DTI integrity were not uncovered in previous research. Our results showed that DTI assessment of the CST was related to the functional status of the affected hand at 6 months after onset. Previous studies also showed that CST integrity, as identified by DTI, predicted restoration of hand function (10, 13, 17). In cerebral palsy, CST recovery reflected clinical improvement after rehabilitative treatment in hemiplegic pediatric patients (28). Taken together, these findings indicate that CST integrity, as assessed by DTI, is related to the functional status of the hand in patients with chronic stroke.

We analyzed DTI integrity using FN, FA (mid-pons), and FA (pontomedullary junction) values using an established method (10, 22, 27). Among these, the mid-pons FA value was more strongly related to hand function than were the others. We suggest that the normalized mid-pons FA values might be more useful that the pontomedullary junction FN or FA values. Among FN, FA (mid-pons), and FA (pontomedullary junction), the DTI value of the normalized FA (mid-pons) might best predict the long-term outcome of hand function in patients with stroke.

A few limitations of our study should be noted. First, we used a cross-sectional design; we did not collect tensor images in the early stage, so changes in DTI over time were not investigated. Second, we classified only three groups according to clinical hand function. The lack of quantitative, well-validated tools for assessing hand function, ranging from no motion to normal motion, limited our ability to evaluate outcomes. Our previous research used the upper limb motor score on the Fugl–Meyer assessment for quantitative analysis (5, 6, 23). This assessment included all upper limb functions. Therefore, the functional status might be overestimated in cases with a non-functional hand. Another study reported that the Fugl–Meyer motor score may be preferable for stroke survivors with lower levels of ability based on these limitations (29). To overcome this methodological limitation, we recruited 44 patients with a first-ever stroke, supratentorial lesion, and moderate to severe hemiplegia. This allowed the descriptive power of DTI integrity to reflect hand function at 6 months after stroke.

In conclusion, the mid-pons FA values reflected CST integrity, and these values at 6 months after stroke onset appear to correspond to full recovery of the functional status of the hand. These results will be useful for the prediction of functional restoration of the hand and will contribute to our understanding of the functional prognosis of stroke.

## Ethics Statement

The study protocol was reviewed and approved by the Institutional Review Board of Catholic University, College of Medicine (Registry No. VC18RESI0048); the need for informed consent was waived by the board.

## Author Contributions

YJY contributed to the concept, results analysis, and manuscript drafting. JWK contributed to results analysis and data acquisition. JSK and BYH provided the subjects and contributed to the manuscript review. KBL contributed to the data acquisition. SHL contributed to the concept, funding acquisition, results analysis, and to drafting, reviewing and finalizing the manuscript.

### Conflict of Interest Statement

The authors declare that the research was conducted in the absence of any commercial or financial relationships that could be construed as a potential conflict of interest.
